# Supplementary material for: Impact of a collaborative model on community clinician confidence in child and adolescent mental health care, wellbeing, and access to child psychiatry expertise
Source: PLoS One. 2024 Sep 23;19(9):e0310377. doi: 10.1371/journal.pone.0310377 (PMC11419376; doi:10.1371/journal.pone.0310377)
Supplement: S1 Appendix — (PDF) [file pone.0310377.s001.pdf]

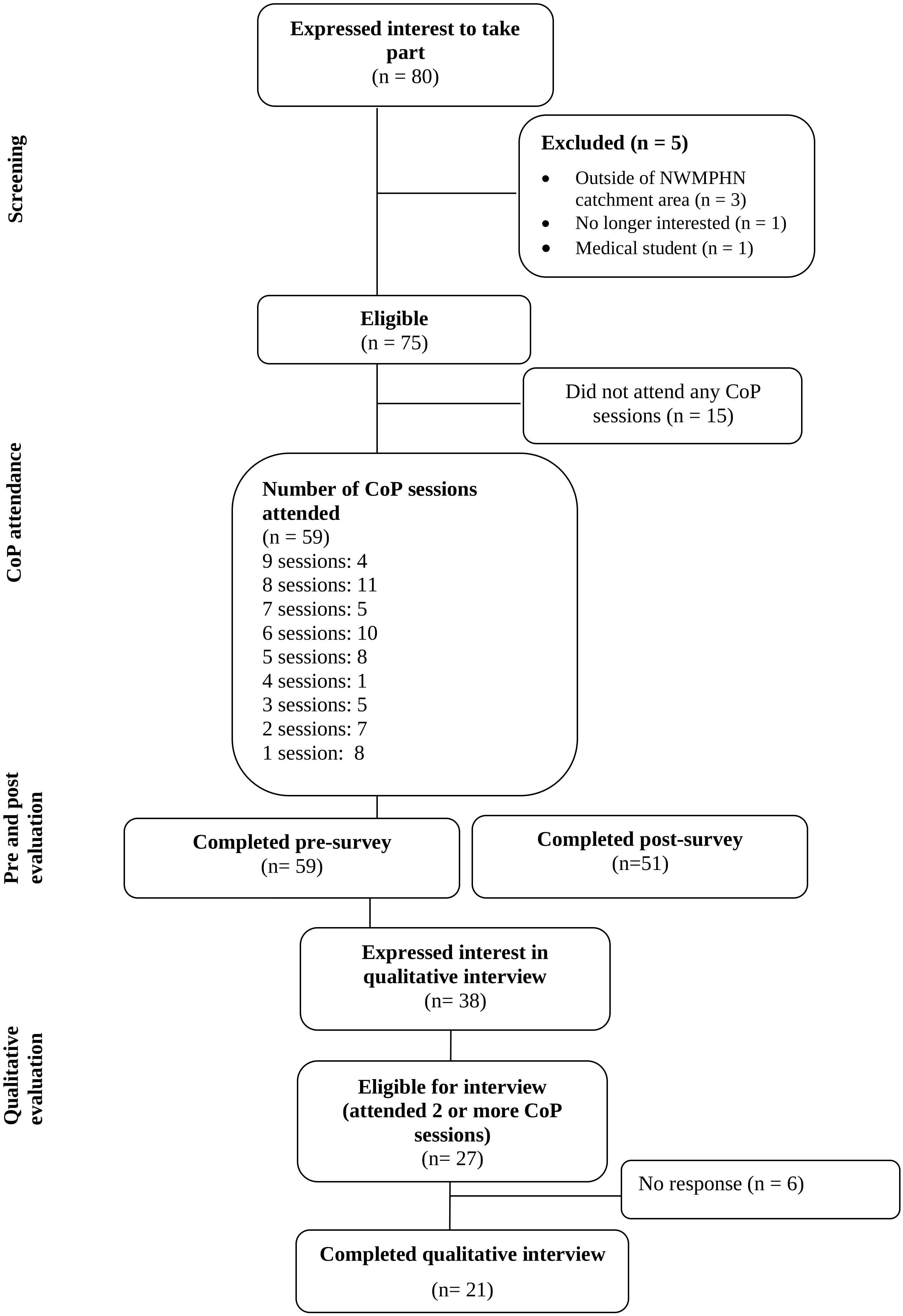
Screening
Expressed interest to take part
(n = 80)
Excluded (n = 5)
Outside of NWMPHN catchment area (n = 3)
No longer interested (n = 1)
Medical student (n = 1)
Eligible
(n = 75)
CoP attendance
Did not attend any CoP sessions (n = 15)
Number of CoP sessions attended
(n = 59)
9 sessions: 4
8 sessions: 11
7 sessions: 5
6 sessions: 10
5 sessions: 8
4 sessions: 1
3 sessions: 5
2 sessions: 7
1 session: 8
Pre and post evaluation
Completed pre-survey
(n= 59)
Completed post-survey
(n=51)
Qualitative evaluation
Expressed interest in qualitative interview
(n= 38)
Eligible for interview (attended 2 or more CoP sessions)
(n= 27)
No response (n = 6)
Completed qualitative interview
(n= 21)
