# Supplementary material for: Impact of a collaborative model on community clinician confidence in child and adolescent mental health care, wellbeing, and access to child psychiatry expertise
Source: PLoS One. 2024 Sep 23;19(9):e0310377. doi: 10.1371/journal.pone.0310377 (PMC11419376; doi:10.1371/journal.pone.0310377)
Supplement: S2 Appendix — (PDF) [file pone.0310377.s002.pdf]

Child Mental Health Pilot: A Community of Practice

About This Survey

This survey is about your experiences in providing child mental health care and management for paediatric (< 18 years) patients.

The survey will take about 10-15 minutes to complete

Thank you!

SECTION 1: Clinician Information

So that we can track who completed this survey, please provide your name

____

Please provide the name of your organisation

____

Please provide your email address

____

What is your gender?

◯ Male
◯ Female
◯ Other

What is your professional Role:

◯ General Practitioner
◯ Paediatrician
◯ Psychologist
◯ Other

Please specify

____

How long have you been practicing in this role?

◯ Less than 6 years
◯ 6 to 15 years
◯ More than 15 years

1.6 How many half day clinical sessions do you work per week?

◯ Less than 6 clinical sessions per week
◯ 6 to 10 clinical sessions per week
◯ More than 10 clinical sessions per week

What is the average number of paediatric (0 - 17 years) patients you see per week?

◯ Less than 11 paediatric patients
◯ 11 to 20 paediatric patients
◯ More than 20 paediatric patients

Have you ever had formal training in paediatric mental health?

◯ Yes
◯ No

Please state the formal mental health training you have had

SECTION 2: Paediatric Mental Health Care and Services
The following sections relates to your perspectives on, and experiences of, paediatric mental health care and services for infants, children and adolescents.
Please read each item, and tick the box that best describes how much you agree with each statement.

For Infants

	Not at all confident	Not very confident	Fairly confident	Completely confident	Not my role
2.1 I know how mental health services are organised	○	○	○	○	○
2.2 I know how to access mental health services	○	○	○	○	○
2.3 I can diagnose mental health conditions	○	○	○	○	○
2.4 I know how to refer for mental health support	○	○	○	○	○

For Children

	Not at all confident	Not very confident	Fairly confident	Completely confident	Not my role
2.5 I know how mental health services are organised	○	○	○	○	○
2.6 I know how to access mental health services	○	○	○	○	○
2.7 I can diagnose mental health conditions	○	○	○	○	○
2.8 I know how to refer for mental health support	○	○	○	○	○
2.9 in prescribing first-line psychotropic medication (e.g for anxiety)	○	○	○	○	○
2.10 in prescribing second and third line psychotropic medication	○	○	○	○	○

For Adolescents

	Not at all confident	Not very confident	Fairly confident	Completely confident	Not my role
2.11 I know how mental health services are organised	○	○	○	○	○
2.12 I know how to access mental health services	○	○	○	○	○
2.13 I can diagnose mental health conditions	○	○	○	○	○
2.14 I know how to refer for mental health support	○	○	○	○	○
2.15 in prescribing first line psychotropic medication (e.g for anxiety)	○	○	○	○	○
2.16 in prescribing second and third line psychotropic medication	○	○	○	○	○

2.2 How important are each of the following personal factors in your decision to refer a child/adolescent to mental health services ?

	Very unimportant	Somewhat unimportant	Somewhat important	Very important
a. I do not have enough knowledge about a specific child's mental health condition	○	○	○	○
b. I have no experience in treating or providing ongoing mental health management of a specific child's condition	○	○	○	○
c. I do not feel comfortable caring for a child with a complex mental health condition	○	○	○	○
d. I do not feel confident in reassuring parents that they do not need to seek a second opinion	○	○	○	○

SECTION 3: Paediatric Mental Health Management
The following sections relate to your confidence in managing non-pharmacological and pharmacological mental health problems for infants, children and adolescents. Please select box that best describes your confidence as a clinician.

3.1 How confident are you in the non-pharmacological management of:

	Not at all confident	Not very confident	Fairly confident	Completely confident	Not my role
Infant regulatory disorders (eating, sleeping, feeding problems)	○	○	○	○	○
Infant attachment disorders	○	○	○	○	○

3.2 For Children, how confident are you in the non-pharmacological management of:

	Not at all confident	Not very confident	Fairly confident	Completely confident	Not my role
a. ADHD	◯	◯	◯	◯	◯
b. Aggression/challenging behaviours	◯	◯	◯	◯	◯
c. Anxiety symptoms/ Generalized Anxiety Disorder/social anxiety	◯	◯	◯	◯	◯
d. Attachment disorders / family relationship difficulties	◯	◯	◯	◯	◯
e. Conduct Disorder	◯	◯	◯	◯	◯
f. Depression	◯	◯	◯	◯	◯
g. Eating disorders- anorexia/bulimia	◯	◯	◯	◯	◯
h. Learning difficulties/Intellectual disability	◯	◯	◯	◯	◯
i. Obsessive-Compulsive Disorder (OCD)	◯	◯	◯	◯	◯
j. Oppositional Defiant Disorder	◯	◯	◯	◯	◯
k. Post-traumatic stress disorder (PTSD)	◯	◯	◯	◯	◯
l. Suicidality	◯	◯	◯	◯	◯
m. Self-harm	◯	◯	◯	◯	◯

3.3 For Adolescents, how confident are you in the non-pharmacological management of:

	Not at all confident	Not very confident	Fairly confident	Completely confident	Not my role
a. ADHD	○	○	○	○	○
b. Aggression/challenging behaviours	○	○	○	○	○
c. Anxiety symptoms/ Generalized Anxiety Disorder/social anxiety	○	○	○	○	○
d. Attachment disorders / family relationship difficulties	○	○	○	○	○
e. Conduct Disorder	○	○	○	○	○
f. Depression	○	○	○	○	○
g. Eating disorders- anorexia/bulimia	○	○	○	○	○
h. Learning difficulties/Intellectual disability	○	○	○	○	○
i. Obsessive-Compulsive Disorder (OCD)	○	○	○	○	○
j. Oppositional Defiant Disorder	○	○	○	○	○
k. Post-traumatic stress disorder (PTSD)	○	○	○	○	○
l. Suicidality	○	○	○	○	○
m. Self-harm	○	○	○	○	○

***Please note: The following relates to the Pharmacological management of paediatric mental health disorders**

3.4 For Children, how confident are you in the pharmacological management of:

	Not at all confident	Not very confident	Fairly confident	Completely confident	Not my role
a. ADHD	◯	◯	◯	◯	◯
b. Aggression/challenging behaviours	◯	◯	◯	◯	◯
c. Anxiety symptoms/ Generalized Anxiety Disorder/social anxiety	◯	◯	◯	◯	◯
d. Attachment disorders / family relationship difficulties	◯	◯	◯	◯	◯
e. Conduct Disorder	◯	◯	◯	◯	◯
f. Depression	◯	◯	◯	◯	◯
g. Eating disorders-anorexia/bulimia	◯	◯	◯	◯	◯
h. Learning difficulties/Intellectual disability	◯	◯	◯	◯	◯
i. Obsessive-Compulsive Disorder (OCD)	◯	◯	◯	◯	◯
j. Oppositional Defiance Disorder	◯	◯	◯	◯	◯
k. Post-traumatic stress disorder (PTSD)	◯	◯	◯	◯	◯
l. Suicidality	◯	◯	◯	◯	◯
m. Self-harm	◯	◯	◯	◯	◯

3.5 For Adolescents, how confident are you in the pharmacological management of:

	Not at all confident	Not very confident	Fairly confident	Completely confident	Not my role
a. ADHD	◯	◯	◯	◯	◯
b. Aggression/challenging behaviours	◯	◯	◯	◯	◯
c. Anxiety symptoms/ Generalized Anxiety Disorder/social anxiety	◯	◯	◯	◯	◯
d. Attachment disorders / family relationship difficulties	◯	◯	◯	◯	◯
e. Conduct Disorder	◯	◯	◯	◯	◯
f. Depression	◯	◯	◯	◯	◯
g. Eating Disorders- anorexia/bulimia	◯	◯	◯	◯	◯
h. Learning difficulties	◯	◯	◯	◯	◯
i. Obsessive-Compulsive Disorder (OCD)	◯	◯	◯	◯	◯
j. Oppositional Defiant Disorder (ODD)	◯	◯	◯	◯	◯
k. Post-traumatic stress disorder (PTSD)	◯	◯	◯	◯	◯
l. Suicidality	◯	◯	◯	◯	◯
m. Self-harm	◯	◯	◯	◯	◯

Section 4: Clinician Interviews
At the end of this pilot, we are interested in learning more about your experiences as a clinician during this community of practice model. Dr Elise Dabaco (paediatrician in training) will be leading this extra part of the pilot with the aim to conduct clinician interviews by telephone in July.

Please tick the box below if you would like to be contacted to learn more about taking part in this interview. This does NOT mean that you must take part - only that you want to hear more! Interviews will be conducted at a mutually agreed time.

◯ Yes, I wish to be contacted regarding the clinician interviews in July.
◯ No

Section 5: Further Comments
Please provide any further comments about caring for paediatric mental health patients below
