# Supplementary material for: Impact of a collaborative model on community clinician confidence in child and adolescent mental health care, wellbeing, and access to child psychiatry expertise
Source: PLoS One. 2024 Sep 23;19(9):e0310377. doi: 10.1371/journal.pone.0310377 (PMC11419376; doi:10.1371/journal.pone.0310377)
Supplement: S3 Appendix — (PDF) [file pone.0310377.s003.pdf]

APPENDIX B - CLINICIAN POST SURVEY

Child Mental Health Pilot: Follow-up Survey

About This Survey

This survey is about your experiences of the Community of Practice in providing child mental health care and management for paediatric (< 18 years) patients.

The survey will take about 10-15 minutes to complete

Thank you!

SECTION 2: Paediatric Mental Health Care and Services
The following sections relates to your perspectives on, and experiences of, paediatric mental health care and services for children and adolescents.
Please read each item, and tick the box that best describes how much you agree with each statement.

For Children

	Not at all confident	Not very confident	Fairly confident	Completely confident	Not my role
2.5 I know how mental health services are organised	○	○	○	○	○
2.6 I know how to access mental health services	○	○	○	○	○
2.7 I can diagnose mental health conditions	○	○	○	○	○
2.8 I know how to refer for mental health support	○	○	○	○	○
2.9 in prescribing first-line psychotropic medication (e.g for anxiety)	○	○	○	○	○
2.10 in prescribing second and third line psychotropic medication	○	○	○	○	○

For Adolescents

	Not at all confident	Not very confident	Fairly confident	Completely confident	Not my role
2.11 I know how mental health services are organised	○	○	○	○	○
2.12 I know how to access mental health services	○	○	○	○	○
2.13 I can diagnose mental health conditions	○	○	○	○	○
2.14 I know how to refer for mental health support	○	○	○	○	○
2.15 in prescribing first line psychotropic medication (e.g for anxiety)	○	○	○	○	○
2.16 in prescribing second and third line psychotropic medication	○	○	○	○	○

2.2 How important are each of the following personal factors in your decision to refer a child/adolescent to mental health services ?

	Very unimportant	Somewhat unimportant	Somewhat important	Very important
a. I do not have enough knowledge about a specific child's mental health condition	○	○	○	○
b. I have no experience in treating or providing ongoing mental health management of a specific child's condition	○	○	○	○
c. I do not feel comfortable caring for a child with a complex mental health condition	○	○	○	○
d. I do not feel confident in reassuring parents that they do not need to seek a second opinion	○	○	○	○

SECTION 3: Paediatric Mental Health Management
The following sections relate to your confidence in managing non-pharmacological and pharmacological mental health problems for children and adolescents. Please select box that best describes your confidence as a clinician.

3.2 For Children, how confident are you in the non-pharmacological management of:

	Not at all confident	Not very confident	Fairly confident	Completely confident	Not my role
b. Aggression/challenging behaviours	○	○	○	○	○
c. Anxiety symptoms/ Generalized Anxiety Disorder/social anxiety	○	○	○	○	○
d. Attachment disorders / family relationship difficulties	○	○	○	○	○
e. Conduct Disorder	○	○	○	○	○
f. Depression	○	○	○	○	○
g. Eating disorders- anorexia/bulimia	○	○	○	○	○
i. Obsessive-Compulsive Disorder (OCD)	○	○	○	○	○
j. Oppositional Defiant Disorder	○	○	○	○	○
k. Post-traumatic stress disorder (PTSD)	○	○	○	○	○
l. Suicidality	○	○	○	○	○
m. Self-harm	○	○	○	○	○

3.3 For Adolescents, how confident are you in the non-pharmacological management of:

	Not at all confident	Not very confident	Fairly confident	Completely confident	Not my role
b. Aggression/challenging behaviours	◯	◯	◯	◯	◯
c. Anxiety symptoms/ Generalized Anxiety Disorder/social anxiety	◯	◯	◯	◯	◯
d. Attachment disorders / family relationship difficulties	◯	◯	◯	◯	◯
e. Conduct Disorder	◯	◯	◯	◯	◯
f. Depression	◯	◯	◯	◯	◯
g. Eating disorders- anorexia/bulimia	◯	◯	◯	◯	◯
i. Obsessive-Compulsive Disorder (OCD)	◯	◯	◯	◯	◯
j. Oppositional Defiant Disorder	◯	◯	◯	◯	◯
k. Post-traumatic stress disorder (PTSD)	◯	◯	◯	◯	◯
l. Suicidality	◯	◯	◯	◯	◯
m. Self-harm	◯	◯	◯	◯	◯

***Please note: The following relates to the Pharmacological management of paediatric mental health disorders**

3.4 For Children, how confident are you in the pharmacological management of:

	Not at all confident	Not very confident	Fairly confident	Completely confident	Not my role
b. Aggression/challenging behaviours	○	○	○	○	○
c. Anxiety symptoms/ Generalized Anxiety Disorder/social anxiety	○	○	○	○	○
d. Attachment disorders / family relationship difficulties	○	○	○	○	○
e. Conduct Disorder	○	○	○	○	○
f. Depression	○	○	○	○	○
g. Eating disorders- anorexia/bulimia	○	○	○	○	○
i. Obsessive-Compulsive Disorder (OCD)	○	○	○	○	○
j. Oppositional Defiance Disorder	○	○	○	○	○
k. Post-traumatic stress disorder (PTSD)	○	○	○	○	○
l. Suicidality	○	○	○	○	○
m. Self-harm	○	○	○	○	○

3.5 For Adolescents, how confident are you in the pharmacological management of:

	Not at all confident	Not very confident	Fairly confident	Completely confident	Not my role
b. Aggression/challenging behaviours	◯	◯	◯	◯	◯
c. Anxiety symptoms/ Generalized Anxiety Disorder/social anxiety	◯	◯	◯	◯	◯
d. Attachment disorders / family relationship difficulties	◯	◯	◯	◯	◯
e. Conduct Disorder	◯	◯	◯	◯	◯
f. Depression	◯	◯	◯	◯	◯
g. Eating Disorders-anorexia/bulimia	◯	◯	◯	◯	◯
i. Obsessive-Compulsive Disorder (OCD)	◯	◯	◯	◯	◯
j. Oppositional Defiant Disorder (ODD)	◯	◯	◯	◯	◯
k. Post-traumatic stress disorder (PTSD)	◯	◯	◯	◯	◯
l. Suicidality	◯	◯	◯	◯	◯
m. Self-harm	◯	◯	◯	◯	◯

Section 4: About the Community of Practice model

Thinking about the Community of Practice sessions you have attended over the last 5 months, please complete the following questions that best describe your experience.

4.1 What was the best thing about this community of practice model?

4.2 What was the worst thing about this community of practice model?

4.3 What could make this community of practice model better?

4.4 Would you recommend this community of practice to other clinicians?

◯ Yes
◯ No

4.5 This Community of Practice model ran for a total of 5 months (10 sessions). What do you think is an ideal number of sessions for a Community of Practice in child and adolescent mental health? Please record below and explain why.

4.6 Have you used any of the Community of Practice resources in your practice?

◯ Yes
◯ No

Which resources have you used?

4.7 As a result of the Community of Practice, have you formed any new connections with the participating clinicians?

◯ Yes
◯ No

4.8 What could we do to support you to maintain your new areas of learning?

☐ Run booster sessions
☐ Establish an online practitioner network
☐ Collate existing session resources on a website/email
☐ Other, please explain below

Please explain

4.9 Do you feel confident in informally supporting colleagues with what you have learnt?

◯ Yes
◯ No

If no , what might make you feel confident to be a go to person in your service or local network? Please explain

4.10 Would you be interested in deepening your skill level (e.g. to participate in a train the trainer course) to more formally support colleagues?

◯ Yes
◯ No
◯ Maybe

4.11 If the Community of Practice model were to continue, how would you like this to happen? What ideas do you have to make it sustainable?

4.12 Do you agree to have your contact details (name, profession, email and phone number) shared with the group?

◯ Yes- phone and email
◯ Yes- email only
◯ No

4.13 Any further comments about this community of practice model?
