# Supplementary material for: Impact of a collaborative model on community clinician confidence in child and adolescent mental health care, wellbeing, and access to child psychiatry expertise
Source: PLoS One. 2024 Sep 23;19(9):e0310377. doi: 10.1371/journal.pone.0310377 (PMC11419376; doi:10.1371/journal.pone.0310377)
Supplement: S4 Appendix — (PDF) [file pone.0310377.s004.pdf]

APPENDIX C- PARTICPANT INFORMATION FORM

HREC Project Number: 73998

Full Name of Project: Clinician Perspectives on a Community of Practice Model for Child Mental Health: A Qualitative Study

Principal Researcher: Dr. Elise D'Abaco (BBiomed, MD, MSc, MMed (paediatric medicine))

Version Number: 1.2 **Version Date:** 03/05/2021

Dear [participant name],

We are inviting you to be involved in a qualitative study as you have taken part in the Community of Practice for Child Mental Health. We want to hear more about your experience of the Community of Practice Model.

At the completion of the Community of Practice sessions, we would like to undertake a recorded interview over the phone with you. The interview includes four open ended questions and is expected to last approximately 30 minutes. We intend to record the interviews to facilitate ease of data analysis. You may decline to be recorded, and field notes can be made instead during the interview. You can nominate a time that suits you to complete the interview and may withdraw at any point if needed.

The data obtained will remain anonymous and stored on the network drive on a password protected computer which can only be accessed by the research team. Written notes will be securely stored in a locked filing cabinet held at Royal Children's Hospital. You can withdraw from the project at any time for any reason. If you withdraw from the project, we will use any information already collected unless you tell us not to. You can request to review a copy of the transcript of your interview prior to data analysis or publication, via email (elisedabaco@gmail.com). Requests must be submitted prior to 1st August 2021. We will endeavour to have these sent within 2-3 days of the request being made.

Using these interviews, we aim to describe the experience of clinicians involved in the Community of Practice and whether their involvement changed their clinical practice with respect to diagnosis, management and referral of child mental health conditions. We are also keen to explore whether the CoP Model had a positive impact on the mental health of the clinicians. In the unlikely event that any participants become distressed throughout the course of the interview, we will terminate the interview, provide psycho-social support, follow-up phone contact and initiate any referrals if required.

If the Community of Practice for Child Mental Health is found to be successful, further investment in this model may ensure that it becomes a cornerstone of our public mental health services, in response to rising child mental health presentations.

If you have any questions about the project or wish to follow-up on the outcomes of the study, you can contact us via phone (0439 351 268) or send an email (elisedabaco@gmail.com).

Thank you very much for your time.

Yours sincerely

Dr Elise D'Abaco
Advanced Trainee in General Paediatrics

You can contact the Director of Research Ethics & Governance at The Royal Children's Hospital Melbourne if you:

- have any concerns or complaints about the project
- are worried about your rights as a research participant
- would like to speak to someone independent of the project.

The Director can be contacted by telephone on (03) 9345 5044.
