# Supplementary material for: Impact of a collaborative model on community clinician confidence in child and adolescent mental health care, wellbeing, and access to child psychiatry expertise
Source: PLoS One. 2024 Sep 23;19(9):e0310377. doi: 10.1371/journal.pone.0310377 (PMC11419376; doi:10.1371/journal.pone.0310377)
Supplement: S5 Appendix — (PDF) [file pone.0310377.s005.pdf]

APPENDIX D - QUALITATIVE INTERVIEW GUIDE

Interview Guide: Health Practitioners

Name: ____ **Date & Time:** _________________________________

Study ID: ______________________________________ **Setting:** ______________________________________

Profession: ____________________________________

Interviewer Initial: ______________________________

SECTION 1 – Experience of the Community of Practice Model

Question	Question Complete
1. Can you describe your experience of the Community of Practice Model?<br>a. We are looking for positives and negatives, there is no wrong answer	☐

SECTION 2 – Feedback

Question	Question Complete
2. Do you have any feedback on the Community of Practice Model?<br>a. What worked well? (*What was the best or most rewarding aspect?)*<br>b. What was challenging? (*What didn't work well?)*<br>*c.* How could it be improved? (*If you were going to run this model with another group, what would you do differently?)*<br>d. What didn't you learn that you expected to learn? (E.g., knowledge, skill or capability)<br>i. Why didn't you learn it? (E.g., was the content not included and/ or was it difficult to learn in a CoP)	☐

SECTION 3 – Impact on Clinical Practice

Question	Question Complete
3. Has your involvement in the CoP Model changed your clinical practice?<br>a. If so, which domains of clinical practice were affected (diagnosis, management or referral)?<br>*b. Can you give me some examples of how your practice has changed?*<br>*c. If you changed to your practice, what did you find?*	☐

SECTION 4 – Impact on Clinician Mental Health

Question	Question Complete
4. In what ways, if any, has the CoP Model impacted your own mental health?	☐

SECTION 5 – Final Comments

Question	Question Complete
5. Do you have anything else to add?	☐
