# Supplementary material for: Impact of a collaborative model on community clinician confidence in child and adolescent mental health care, wellbeing, and access to child psychiatry expertise
Source: PLoS One. 2024 Sep 23;19(9):e0310377. doi: 10.1371/journal.pone.0310377 (PMC11419376; doi:10.1371/journal.pone.0310377)
Supplement: S6 Appendix — (PDF) [file pone.0310377.s006.pdf]

APPENDIX E – SECONDARY CONSULTATION RECORD LOG

RCH Consultation Service logbook						
	How contacted	Who contacted	Patient gender/ age	Type of consultation	Reason for consultation (tick as many as relevant)	Outcome
Patient 1	☐ Phone<br>☐ Email<br>☐ Other: ___________	☐ GP<br>☐ Paediatrician<br>☐Psychologist<br>☐ Other	☐ Male<br>☐ Female<br>☐ Other<br>Patient age: ______________	☐ Primary Consultation<br>☐ Secondary Consultation	☐ Medication advice<br>☐ Diagnostic review<br>☐ Treatment resistance needing comprehensive MH review	☐ sent back to referrer<br>☐ Referred to CAMHS<br>☐ Other: __________
Patient 2	☐ Phone<br>☐ Email<br>☐ Other: _________	☐ GP<br>☐ Paediatrician<br>☐ Psychologist<br>☐ Other	☐ Male<br>☐ Female<br>☐ Other<br>Patient age: ______________	☐ Primary Consultation<br>☐ Secondary Consultation	☐ Medication advice<br>☐ Diagnostic review<br>☐ Treatment resistance needing comprehensive MH review	☐ sent back to referrer<br>☐ Referred to CAMHS<br>☐ Other: __________
Patient 3	☐ Phone<br>☐ Email<br>☐ Other: _________	☐ GP<br>☐ Paediatrician<br>☐ Psychologist<br>☐Other	☐ Male<br>☐ Female<br>☐ Other<br>Patient age: ______________	☐ Primary Consultation<br>☐ Secondary Consultation	☐ Medication advice<br>☐ Diagnostic review<br>☐ Treatment resistance needing comprehensive MH review	☐ sent back to referrer<br>☐ Referred to CAMHS<br>☐ Other: __________
Patient 4	☐ Phone<br>☐ Email<br>☐ Other: _________	☐ GP<br>☐ Paediatrician<br>☐ Psychologist<br>☐Other	☐ Male<br>☐ Female<br>☐ Other<br>Patient age: ______________	☐ Primary Consultation<br>☐ Secondary Consultation	☐ Medication advice<br>☐ Diagnostic review<br>☐ Treatment resistance needing comprehensive MH review	☐ sent back to referrer<br>☐ Referred to CAMHS<br>☐ Other: __________
Patient 5	☐ Phone<br>☐ Email<br>☐ Other: _________	☐ GP<br>☐ Paediatrician<br>☐ Psychologist<br>☐Other	☐ Male<br>☐ Female<br>☐ Other<br>Patient age: ______________	☐ Primary Consultation<br>☐ Secondary Consultation	☐ Medication advice<br>☐ Diagnostic review<br>☐ Treatment resistance needing comprehensive MH review	☐ sent back to referrer<br>☐ Referred to CAMHS<br>☐ Other: __________
Patient 6	☐ Phone<br>☐ Email<br>☐ Other: _________	☐ GP<br>☐ Paediatrician<br>☐ Psychologist<br>☐Other	☐ Male<br>☐ Female<br>☐ Other<br>Patient age: ______________	☐ Primary Consultation<br>☐ Secondary Consultation	☐ Medication advice<br>☐ Diagnostic review<br>☐ Treatment resistance needing comprehensive MH review	☐ sent back to referrer<br>☐ Referred to CAMHS<br>☑ Other: __________

APPENDIX E – SECONDARY CONSULTATION RECORD LOG

Patient 7	☐ Phone<br>☐ Email<br>☐ Other: ___________	☐ GP<br>☐ Paediatrician<br>☐Psychologist<br>☐Other ___________	☐ Male ☐ Female ☐ Other<br>Patient age: ___________	☐ Primary Consultation<br>☐ Secondary Consultation	☐ Medication advice<br>☐ Diagnostic review<br>☐ Treatment resistance needing comprehensive MH review	☐ sent back to referrer<br>☐ Referred to CAMHS<br>☐ Other: ___________
Patient 8	☐ Phone<br>☐ Email<br>☐ Other: ___________	☐ GP<br>☐ Paediatrician<br>☐Psychologist<br>☐ Other	☐ Male<br>☐ Female<br>☐ Other<br>Patient age: ___________	☐ Primary Consultation<br>☐ Secondary Consultation	☐ Medication advice<br>☐ Diagnostic review<br>☐ Treatment resistance needing comprehensive MH review	☐ sent back to referrer<br>☐ Referred to CAMHS<br>☐ Other: ___________
Patient 9	☐ Phone<br>☐ Email<br>☐ Other: ___________	☐ GP<br>☐ Paediatrician<br>☐ Psychologist<br>☐ Other	☐ Male<br>☐ Female<br>☐ Other<br>Patient age: ___________	☐ Primary Consultation<br>☐ Secondary Consultation	☐ Medication advice<br>☐ Diagnostic review<br>☐ Treatment resistance needing comprehensive MH review	☐ sent back to referrer<br>☐ Referred to CAMHS<br>☐ Other: ___________
Patient 10	☐ Phone<br>☐ Email<br>☐ Other: ___________	☐ GP<br>☐ Paediatrician<br>☐ Psychologist<br>☐Other	☐ Male<br>☐ Female<br>☐ Other<br>Patient age: ___________	☐ Primary Consultation<br>☐ Secondary Consultation	☐ Medication advice<br>☐ Diagnostic review<br>☐ Treatment resistance needing comprehensive MH review	☐ sent back to referrer<br>☐ Referred to CAMHS<br>☐ Other: ___________
Patient 11	☐ Phone<br>☐ Email<br>☐ Other: ___________	☐ GP<br>☐ Paediatrician<br>☐ Psychologist<br>☐Other	☐ Male<br>☐ Female<br>☐ Other<br>Patient age: ___________	☐ Primary Consultation<br>☐ Secondary Consultation	☐ Medication advice<br>☐ Diagnostic review<br>☐ Treatment resistance needing comprehensive MH review	☐ sent back to referrer<br>☐ Referred to CAMHS<br>☐ Other: ___________

Primary Consultation: seeing patient for consult, seeing patient for co-consult in-person or telehealth

Secondary Consultation: advice only by phone or email
