# Supplementary material for: Impact of a collaborative model on community clinician confidence in child and adolescent mental health care, wellbeing, and access to child psychiatry expertise
Source: PLoS One. 2024 Sep 23;19(9):e0310377. doi: 10.1371/journal.pone.0310377 (PMC11419376; doi:10.1371/journal.pone.0310377)
Supplement: Attachment — Submitted filename: PONE-D-23-1439- Response to Reviewers.docx [file pone.0310377.s007.docx]

10^th^ October 2023

Lea Sacca

Academic editor

PLOS ONE

Dear Lea

Re: PONE-D-23-1439

Many thanks for the opportunity to revise our paper which we believe is considerably improved thanks to reviewer comments.

We have now clearly stated our key aims (6 aims in all) for this paper and ensured that our aims align with the methodology and results. In doing this, we have deleted a small section of the original paper that focused on clinician self-reported factors that may affect their decision to refer a patient (summarized in the original table 2). We felt that these 4 factors were not particularly informative and lead to unnecessary lengthening of the paper, but we would be happy to re-instate them if you wish.

Kind regards,

Professor Harriet Hiscock MB BS, FRACP, MD, FAAHMS, GAICD

On behalf of the co-authors

| **Reviewer comment** | **Response** |
| --- | --- |
| **Reviewer #1: Abstract** 1. Specificity of objectives: The objectives mentioned are quite general ("confidence, competence, and well-being of community clinicians"), and it might be beneficial to provide more specific details about what aspects of confidence, competence, and well-being are being measured. This would give reviewers a clearer understanding of the study's focus.  2. Lack of background: The abstract lacks background information about the current state of child and adolescent mental health care, the challenges faced by community clinicians, and the need for collaborative models like COMPASS. Providing a brief context would help reviewers understand the significance of the study.  3. Methods and measures: The abstract briefly mentions pre-post surveys and semi-structured interviews as methods, but it doesn't elaborate on the specific scales or tools used for assessing confidence, competence, and well-being. Including details about the measurement tools and their validity/reliability would enhance the methodological transparency.  4. Qualitative analysis approach: The description of the qualitative analysis is quite brief ("inductive approach" and "content analysis"). Elaborating on the process of qualitative data analysis, including coding strategies, inter-coder reliability, and how themes are being generated, would provide a clearer picture of the rigor in the qualitative component.  5. Outcomes of consultations: The abstract mentions a child psychiatrist's snapshot of consultations without explaining the purpose of this snapshot or how it contributes to the study's objectives. Clarifying how this data will be used and what insights it aims to provide would be beneficial. | 1. We have now clarified our objectives in the Abstract and Methods as follows: “ *We aimed to assess the impact of COMPASS on community clinician self-reported confidence in: managing common child and adolescent mental health disorders (Aim 1, primary outcome); navigating the mental health care system (i.e. knowing how services are organised, accessed, and how to refer patients, Aim 2); diagnosing conditions (Aim 3); prescribing psychotropic medications (Aim 4) as well as the impact on, clinician practice and wellbeing (Aim 5) and outcomes of patients referred by COMPASS clinicians to the child psychiatrist (Aim 6)*.” 2. We have now added this detail in the Abstract as follows: “*The COVID-19 pandemic was associated with an increase in child and adolescent mental health disorders, with subsequent worsening of patient access to specialist mental health care. Clinicians working in the community were faced with increased demands to diagnose and manage pediatric mental health disorders, without always having the confidence and knowledge to do so. We therefore developed COnnecting Mental-health PAediatric Specialists and community Services (COMPASS) - a collaborative model designed to upskill community clinicians in child and adolescent mental health care and provide them with better access to child and adolescent psychiatry expertise*.” 3. The measures were study-designed and based on previous work by author HH assessing practitioner’s change in confidence. This has been clarified in the Abstract and elaborated on in the Methods on pages 15-16. 4. We have now elaborated as follows in the Abstract: “*We adopted an inductive approach to the qualitative analysis using the Framework Method. This involved selecting five random transcripts which were double coded and categorized, to generate an initial framework against which all subsequent transcripts were analysed. Themes and subthemes were generated from the data set, by reviewing the matrix and making connections within and between clinicians, codes and categories*.” 5. This is now clarified as per Aim 6 above. |
| **Introduction** 1. Clarity and focus: While the introduction covers a wide range of relevant information, it could benefit from greater focus. The introduction should clearly state the research problem, objectives, and research questions. The reader should be able to understand the main purpose of the study from the introduction alone.  2. Citation usage: The introduction relies heavily on citations, which is good for providing evidence but can become overwhelming. It's important to strike a balance between providing evidence and maintaining a coherent narrative. Some information might be better summarized instead of directly quoting from sources.  3. Transition and flow: The introduction lacks smooth transitions between the different sections. Each paragraph should naturally lead to the next, guiding the reader through the argument. Consider using transitional phrases to connect ideas and improve the overall flow.  4. Research gap: While the introduction mentions the lack of evaluated models in Australia, it could further emphasize the research gap in the existing literature. What specific aspects of the current knowledge are insufficient? How will the proposed study address these gaps?  5. Theoretical framework: It would be beneficial to include a brief overview of the theoretical framework that underpins the study. What theoretical perspectives are guiding the research? This will help readers understand the context within which the study is situated.  6. Hypotheses/research questions: The introduction should clearly present the hypotheses or research questions that the study aims to address. This provides readers with a clear roadmap for what the study intends to investigate.  7. Significance and implications: Highlight the significance of the study in addressing the identified gaps. What are the potential implications of the study's findings for the field of child and adolescent mental health, clinical practice, and policy development?  8. Methodology preview: While the introduction does briefly mention the study's implementation and evaluation of the COMPASS model, a clearer preview of the research methodology would be valuable. This could include a brief overview of the study design, data collection methods, and analytical approaches.  9. Engagement with existing literature: While the introduction references a few statistics and the lack of evaluated models, it could engage more deeply with the existing literature on community-based mental health care and the challenges faced by clinicians. This will help position the study within the broader academic discourse. | 1. We have now stated the research problem on page 8 “However*, many community clinicians report they have inadequate training in pediatric mental health and require more professional development opportunities to improve their confidence in diagnosing and managing child and adolescent mental health conditions and to navigate the complex mental health system.* “ and our key aims (as per the Abstract above) now on pages 10 and 11. 2. Thank you – we have endeavoured to delete some text in the Introduction (e.g. on page 5) 3. We have now added an introductory sentence to paragraph 2, altered paragraph 3 to improve transition, and added a 4^th^ paragraph summarizing our Aims and potential impact of COMPASS on clinicians in the community and specialist mental health services. 4. We have now added details of our previous work (reference 13) with clinicians highlighting their need for further training and support on page 8 and on page 10, we have detailed other research gaps in the literature as follows “Further*, it is unknown whether such models impact clinician wellbeing or if they are associated with a reduction in referrals to specialist services* .” 5. We did not have a theoretical framework for this study. Rather, in a response to heightened need during COVID-19 in Melbourne, Australia (the most locked down city in the world), we stood up the COMPASS model and aimed to evaluate its impact on clinician confidence in care and outcomes of patients referred to the child psychiatrist, as per our Aims outlined above. 6. We have now added our Aims on page 11, as per the Aims in the Abstract outlined above. 7. We now state on page 11 the potential significance of COMAPSS as follows; “*If effective, COMPASS could offer an acceptable way to improve capacity of community clinicians to provide pediatric mental health care, navigate the mental health system and improve their access to child psychiatry expertise. This in turn could reduce the burden on overloaded specialist mental health services and provide policy makers with scalable solutions to improve workforce capability.*” 8. We have now briefly discussed our methods in the Introduction on page 11 as follows: “*Using clinician pre-post surveys, in-depth interviews and child psychiatrist consultation data, we aimed to determine whether COMPASS aimed could increase clinician self-reported confidence in….”* 9. We have now expanded on the challenges faced by Australian clinicians from our previous study on pages 8 and 9 as follows: “We *previously interviewed 143 child and adolescent psychiatrists, paediatricians, child psychologists and general practitioners to understand their perspectives on problems and solutions for the Australian paediatric mental health system. Clinicians reported problems with service fragmentation, long wait times, and for GPs and paediatricians - inadequate training in child and adolescent mental health[13]. Solutions included increased access to child psychiatry expertise, mental health training, and co-located multidisciplinary services*.” |
| **Methods/materials** 1. Clarity of procedures: there could be clearer organization and separation of the different stages or components. The description could be broken down into subsections, such as "Recruitment of Participants," "Co-Development of CoP," "Delivery of CoP Sessions," "Qualitative Interviews," etc., to enhance readability and structure.  2. Rationale for method selection: Provide more justification for the chosen methods. Why were CoP sessions and qualitative interviews selected as the main approaches to evaluate the COMPASS model's impact? How do these methods align with the study's objectives and research questions?  3. Sampling and recruitment details: Provide more information about the size and characteristics of the participant pool. How many clinicians were initially approached, and how many participated in the CoP sessions and surveys? How were participants selected for interviews? Including this information provides context for the representativeness of the sample.  4. CoP content and structure: While the content of the CoP sessions is mentioned, there could be more detail about the development process, how the topics were selected, and the rationale behind choosing those specific areas.  5. Ethical considerations and informed consent: While the ethics approval is mentioned, it would be beneficial to elaborate on the ethical considerations taken into account during the study. Describe the steps taken to ensure participant confidentiality and how informed consent was obtained, particularly for both surveys and interviews.  6. Data collection and analysis: Elaborate on the process of data collection, management, and analysis. How were surveys administered? How were survey responses coded and analyzed? Similarly, how were qualitative interviews conducted, transcribed, and analyzed?    7. Validity and reliability: Discuss steps taken to ensure the validity and reliability of both the quantitative and qualitative data. This could include measures to ensure consistent administration of surveys, inter-rater reliability for coding qualitative data, and methods to enhance data triangulation.  8. Limitations and potential biases: Address potential limitations and biases in the study design and methods. For instance, what are the limitations of relying on self-reported measures of confidence? These can also be included in the Discussion.  9. Alignment with research objectives: Continuously relate the methods back to the research objectives and questions. Ensure that each method's purpose is clear and directly connected to the study's overall aims.  10. Appendices: References to appendices are made without providing the content of the appendices. While they should be provided separately, consider adding a brief description of what each appendix contains to guide readers. | 1. Thank you for this - we have re-organised the Materials and Methods section as per your suggestion. 2. We now clarify on page 14 the methodology we used for each Aim as follows: “To *assess Aims 1 to 4, we conducted pre- (see Appendix B) and post-CoP (see Appendix c) online surveys. Clinicians who expressed interest in completing an interview in the pre-survey and attended two or more CoP sessions, were eligible for interview (see below). Interviews aimed to understand clinicians’ perceptions of COMPASS and its impact on their wellbeing (Aim 5). We chose qualitative interviews as a methodology to gain a richer, more in-depth understanding of clinician perspectives than that provided by survey data. The child psychiatrist completed a study-designed logbook to record the nature and outcomes of their consultations over a two-week period (Aim 6).”* 3. We have now clarified participant flow as per the following on page 13: “Fifty*-nine of the 75 eligible clinicians who registered to take part in the CoP attended at least one session, with 60% of clinicians attending more than 5 out 10 sessions (see Appendix A – participant flowchart*).” and Appendix A - Participant Flowchart. 4. On page 12 we have added a new section (Co-development of the CoP) and stated: “Clinicians *attended up to two, one-hour co-development sessions in February 2021 to develop the content and structure of the CoP sessions. The first session focused on the key conditions clinicians wanted training in and how, with clinicians selecting common conditions that they saw in their clinical practice and opting for expert summaries on diagnosis and management followed by a case discussion. The second session focussed on how the CoP would run e.g. duration of the CoP sessions, when to conduct them (i.e. choice of during and after hours), and preferred virtual conferencing platform.”* 5. We have further elaborated on ethical considerations on page 18 as follows: “*Due to the low-risk nature of the surveys, participants’ completion of the surveys was approved as implied consent. To ensure confidentiality, any information that connected participant contact details to their online survey was stored securely and separately on a password protected computer and only accessed by SK.*” and on page 19 for the clinician interviews as follows: “*Several steps were taken to ensure confidentiality of participants. Identifying details were removed from the recordings and transcripts, and participants were given an ID number. Electronic data, including interview recordings and transcripts, were stored on the network drive on a password protected computer. At the conclusion of the study, all identifying participant data was destroyed and final study data was stored in a non-identifiable format*.” 6. We have added further detail on surveys on page 15 as follows: “*Clinicians were sent pre and post online surveys with two reminders through REDCap, a secure, web-based application for building and managing online surveys and databases, developed by Vanderbilt University [23, 24]. All clinicians were sent the pre survey one month prior to the commencement of the CoP and the post survey one week following the last CoP session*.” We believe pages 16-17 already contain details about how the qualitative interviews were conducted (i.e. by telephone), transcribed (i.e. verbatim by Rev©) and then analyzed. 7. For the surveys, all data were collected via REDCAP with either fixed response options (e.g. for confidence ranges) or sense checking of responses (e.g. age) before data analysis. For the interviews, member checking was offered to clinicians, but none accepted the offer. We did not perform formal inter-rater reliability but rather the two coding authors double coded 5 random transcripts to create an initial framework, against which all subsequent transcripts were analyzed. 8. We have now added these limitations in the Discussion on page 42 9. We believe we have now done this by stating up front (Abstract and Introduction) our 6 Aims and corresponding methodology to address each aim in the Materials and Methods section. 10. Thank you - we have now done this. |
| **Results** 1. Clarity and structure: While the section is well-organized and divided into themes and subthemes, some of the subthemes could benefit from clearer headings or labels.  2. Data representation: ensure that the data are clearly labeled and explained in the text. For instance, in Table 1, it would be helpful to include a brief description of each characteristic being presented, especially for readers who may not be familiar with the field.  3. Quotation usage: consider providing more context for each quote. Briefly explain who the participant is (e.g., GP, psychologist) and their role, which would add credibility to the qualitative findings.  4. Statistical significance and effect size: When presenting quantitative data, it's important to indicate whether any observed changes are statistically significant and, when possible, provide effect size information. | 1. This has now been done, aligning with each Aim. 2. We have done this for characteristics that may be unclear (eg role duration is now Duration of Practice). 3. This has now been added/expanded for all the quotes. 4. We have now added the following on page 16: “*We considered changes with a p value of 0.05 or less to be statistically significant*.”. We have consulted with our institute’s biostatical department and have confirmed that it is not possible to calculate effect sizes for proportional data (as opposed to continuous data). |
| **Discussion and conclusion** 1. Clear linkage to research objectives and findings: The discussion should start by explicitly linking back to the research objectives or hypotheses stated in the introduction.  2. Causality and study design: It's important to emphasize that the study used a pre-post design, which allows for examining changes within the same group over time but doesn't establish causality. This limitation should be acknowledged, and potential confounding factors discussed. Additionally, the discussion could highlight how future research could incorporate control groups or more rigorous study designs to strengthen causal inferences.  3. Comparative analysis with other models: While the discussion mentions other models like Project TEACH and BHIP, it could provide a more detailed comparative analysis of COMPASS with these models in terms of outcomes, implementation, and potential advantages and disadvantages. This would help readers understand how COMPASS contributes uniquely to the field.  4. Challenges and lessons learned: Beyond facilitation and group dynamics, other challenges that emerged during the implementation of the COMPASS model should be discussed. This could include issues related to participant engagement, technology.  5. Generalizability and external validity: Discuss the generalizability of the findings. How representative is the sample of the broader clinician population? Are there potential biases in the sample that could affect the applicability of the findings to other settings or regions?  6. Implications for policy and practice: discuss how the findings of this study could inform decision-making at both policy and practice levels. How could the COMPASS model be adapted or replicated in other regions or healthcare systems?  7. Limitations of qualitative analysis: The qualitative analysis reached data saturation after analyzing about half of the transcripts. While this is common in qualitative research, acknowledge that data saturation might not capture the full range of perspectives and experiences. Discuss potential areas that might have been missed due to this limitation.  8. Future Directions: Provide concrete suggestions for future research directions. This could include exploring the long-term sustainability of the COMPASS model, evaluating its impact on patient outcomes, or investigating the barriers to wider implementation. | 1. We have now re-vamped the first paragraph of our Discussion to address this. 2. We have now addressed this on page 42 stating “*Potential confounders such as clinicians accessing other MH training to improve confidence or organising their own access to a child psychiatrist cannot be ruled out. Future research could address this by conducting a randomized controlled trial of COMPASS, whereby clinicians are randomly assigned to participate in COMPASS or not. However, it may prove difficult to recruit clinicians to a control group.*” Note, we had already stated “As it was a pre-post design, causality cannot be assumed.” 3. We have now elaborated on similarities/differences between COMPASS and (1) Project TEACH on page 40 and (2) BHIP on page 41. 4. On pages 38 and 39, we have expanded on lessons learned to facilitate participant engagement including need for equal representation by practitioner type, setting clear expectations of what COMPASS is, and effective use of breakout rooms, chat functions etc to encourage participation from all participants. 5. Under our Strengths and Limitations section we have explained “*Clinicians were from a range of disciplines with varying clinical experience suggesting our findings could generalise to a range of healthcare providers. However, clinicians self-selected into COMPASS and are thus likely to represent a group motivated to improve pediatric mental health care.*“ Unfortunately, Melbourne does not have representative data on clinician age, gender, years of practice, number of children seen/week to allow us to determine the extent to which our sample is representative or not. 6. We have added the following sentence to page 44: “*It could be further scaled to other healthcare systems where there is a lack of access to child psychiatry expertise and a need to upskill community clinicians*.” 7. We have now acknowledged this on pages 42-43, saying: ”A*lthough we reached data saturation with our qualitative interviews, data saturation might not capture the full range of perspectives and experiences in the broader clinical community.”* And on page 43 we say: “*Our sample was mostly females, who were relatively experienced clinicians. It may have been useful to hear from less experienced clinicians to better understand whether COMPASS met their needs.”* 8. We now say on page 43: “*We have evaluated COMPASS in its first year of operation and from clinicians’ perspectives only. Future research could evaluate the longer-term impacts on clinicians’ confidence and wellbeing, impacts on patient outcomes, and barriers and enablers to scaling the model*.” |
| **Reviewer 2**  **Title**: Impact of a collaborative model on community clinician confidence and competence in child and adolescent mental health: mixed method analysis  - Why the expression "confidence and competence"? Maybe describe it better in the methodology, or adjust it, as it becomes confusing when reading the title. - I'm not sure if "mixed method analysis" is the best to use, because in terms of statistical methods, the study is limited. It's a study where I work with qualitative and quantitative data | We have edited the title to now read: “Impact of a collaborative model on community clinician confidence in child and adolescent mental health care, wellbeing, and access to child psychiatry expertise.” – to address these concerns and better align with our aims. |
| **Abstract** - Objectives are extensive. Determine a primary objective. - Results: Present the outcome of the association mentioned in the conclusion. | - Objectives: We have now stated our primary objective as follows: “We *aimed to assess the impact of COMPASS on community clinician self-reported confidence in: managing common child and adolescent mental health disorders (Aim 1, primary outcome, )*;…” - Results: we have now added the outcome for Aim 1 |
| **Introduction** - It's extensive and still doesn't clarify well how the Community of Practice functions. Is it a government program, an initiative by someone, funded by someone? Does it still exist, or was it an emergency response during the pandemic? Is COMPASS an example of a Community of Practice?  - The study's objective is mixed with the COMPASS objective. Adjust it. | - Thank you – we have now clarified the nature of COMPASS, how it was set up, who funds it, the fact that it is still running etc. - The study’s aims have now been clearly stated and linked to Methods and Results. |
| **Methods** - Who facilitated the sessions, a trained researcher, a clinical member? - What were the development sessions like? - Please include the ethical approval at the end of the methodology. - Regarding ethical considerations, shouldn't a consent form have been signed? Additionally, for data collected from children and adolescents, shouldn't their guardians have been informed and given consent to participate? If it's secondary data, wouldn't there need to be a specific protocol for secondary data? - Are the data from children and adolescents sourced from a database, medical record collection, or patients treated by the recruited clinicians? - Reorganize the methodology (Design and Sample, Data Collection, Ethical Aspects...) - Page 10, line 156: "Clinician pre and post surveys included demographic items (pre-survey only)." What data? - Was a sample calculation performed? Is the sample size sufficient to make any claims? - The methodology needs to be rewritten and reorganized. It requires information regarding the study design, the data used, and how they were analyzed (including specific statistical tests). I understand that COMPASS is an approach created by the study, so the objective and details of COMPASS should be included in the methodology. -I suggest using a flowchart to describe participant recruitment and attrition. | - A general practitioner acted as the facilitator – now stated on page 13 - On page 12 we have now outlined how the sessions were developed, as per our response to Reviewer 1 - We have now moved the ethical approval information to the end of the Methods and Materials section - On page 18 we now state: “Due *to the low-risk nature of the surveys, participants’ completion of the surveys was approved as implied consent.”* - We did not collect data from children or adolescents in this study nor did we collect secondary data. - We have re-organized Methodology in line with Reviewers 1 and 2 - We now state on page 15: “ *Pre- and post-surveys included demographic items (pre-survey only) including clinician age, gender, years of practice etc (see Table 1)”* - This was an evaluation of a model of care stood up during COVID-19 to meet an urgent clinical demand for access to mental health upskilling of community clinicians and child psychiatry expertise. As such, we did not perform a formal sample size calculation. We have been careful to acknowledge that our findings are associations and that there is a need for an adequately powered RCT (page 42). - We have considerably re-worked the Methodology, by each Aim, and included the data used to address each aim and where relevant (ie for quantitative data only), the accompanying statistical analyses. - We have now done this (see Appendix A) |
| **Results** - Page 12, lines 204-211: This belongs in the methodology. - Tables: Describe all acronyms and symbols used in the captions, even if they are obvious (such as n). - Use lowercase n for sample size. This applies to all figures and tables. - Table 3: Describe statistical significance (*) in the caption. - Figures 1 and 2: Remove the underlining from the figure titles. - Pages 19-20, lines 269-281: This is methodology. - Are the 27 eligible clinicians from the quantitative data among the 59 participants from the quantitative part of the CoP session data, or do you have two different samples? | - Thank you - we have now moved this section - We have now done this - We have now changed to lower case n - This was an error and the asterisk has been removed - We have removed the underlining, moved lines 269-281, and provided a Participant Flowchart (Appendix A) to clarify the sample flow. |
| **Discussion** - Page 415: "As per our aims, the COMPASS model was associated with increased community clinician knowledge of services, confidence in diagnosis and management of common paediatric MH conditions, and reduced referrals to CAMHS." Where is this data in the results? - Describe the study's limitations more comprehensively. | - We have now considerably revised our paper to have discrete aims with corresponding methods and results and have summarized these in the opening paragraph of the Discussion. - As per reviewer 1, we have expanded on the limitations of our study |
