# Supplementary material for: Impact of a collaborative model on community clinician confidence in child and adolescent mental health care, wellbeing, and access to child psychiatry expertise
Source: PLoS One. 2024 Sep 23;19(9):e0310377. doi: 10.1371/journal.pone.0310377 (PMC11419376; doi:10.1371/journal.pone.0310377)
Supplement: Attachment — Submitted filename: Response to Reviewers_PLOS ONE.docx [file pone.0310377.s008.docx]

20 August 2024

Saiendhra Vasudevan Moodley, PhD

Academic Editor

PLOS ONE

Dear Dr Moodley,

Re: PONE-D-23-14369R1

Impact of a collaborative model on community clinician confidence in child and adolescent mental health care, wellbeing, and access to child psychiatry expertise.

I have made your requested changes to our revised manuscript i.e. to:

1. In abstract, please replace your first heading "Objectives" with "Background".

2. Replace all the references to Aims 1-6 throughout the paper and replace these with Objectives 1-6.

Thank you for finally moving this paper on!

Kind regards,

Professor Harriet Hiscock
